# BLAST—bladder lithiasis assessment of surgical treatment in real-world practice. A global prospective multicentre registry by EAU Endourology

**DOI:** 10.1007/s00345-026-06813-3

**Published:** 2026-10-13

**Authors:** Vineet Gauhar, Steffi Kar Kei Yuen, Thomas Herrmann, Jaisukh Kalathia, Amelia Pietropaolo, Khi Yung Fong, Arun Chawla, Tianyu Li, Alexey Martov, Zelimkhan Tokhtiev, Giorgio Mazzon, Amish Mehta, Yash Pamecha, Lin Xiong, Angelo Cormio, Ender Cem Bulut, Nariman Gadzhiev, Sohani Dassanayake, Azizbek Shomarufov, Lim Tze Ying Benjamin, Theodoros Tokas, Gunal Ozgur, Luca Boeri, Yunteng Huang, Yasser Gamil Elnagar, Zijie Chai, Bohan Wang, William Lay Keat Ong, Benedikt Becker, Abdul Haseeb, Lazaros Tzelves, Alba Sierra, Thiago Hota, Mallikarjuna Chiruvella, Deepak Ragoori, Chi-Fai Ng, Daniele Castellani, Bhaskar Somani

**Affiliations:** 1 Department of Urology, Ng Teng Fong General Hospital, Singapore, Singapore; 2 Department of Urology, Asian Institute of Nephrology & Urology, Hyderabad, Telangana India; 3 European Association of Urology Section of Endourology, Arnhem, The Netherlands; 4 Department of Surgery, S.H. Ho Urology Centre, The Chinese University of Hong Kong, Hong Kong, China; 5 Department of Urology, Spital Thurgau AG, Kantonsspital Frauenfeld, Pfaffenholzstrasse, Frauenfeld, Switzerland; 6 Department of Urology and Kidney Transplantation, Fortune Urology Clinic, Botad, India; 7 Department of Urology, University Hospital Southampton NHS Foundation Trust, Southampton, UK; 8 Department of Urology, Singapore General Hospital, Singapore, Singapore; 9 Urology and Renal Transplant, Kasturba Medical College, MAHE, Manipal, Karnataka India; 10 Department of Urology, The First Affiliated Hospital of Guangxi Medical University, Nanning, China; 11 Department of Urology and Andrology of A.I. Burnazyan SRC FMBC, FMBA of Russia, Moscow, Russia; 12 Department of Urology, University College London Hospitals, London, UK; 13 B T Savani Kidney Hospital, Rajkot, India; 14 Pamnani Hospital and Research Center Mandsaur, Mandsaur, India; 15 Division of Urology, Shenzhen Hospital of University of Hong Kong, Shenzhen, China; 16 Urology Unit, Azienda Ospedaliero-Universitaria delle Marche, Ancona, Italy; 17 Department of Urology and Renal Transplantation, Policlinico Riuniti di Foggia, University of Foggia, Foggia, Italy; 18 Department of Urology, School of Medicine, Gazi University, Ankara, Turkey; 19 Department of Urology, Saint Petersburg State University Hospital, Saint Petersburg, Russia; 20 Department of Urology, Tashkent State Medical University (Republican Specialized Scientific and Practical Medical Center of Urology), Tashkent, Uzbekistan; 21 Department of Surgical Disciplines, Kimyo International University in Tashkent, Tashkent, Uzbekistan; 22 Medical School, Central Asian University, Tashkent, Uzbekistan; 23 Department of Urology, Sengkang General Hospital, Singapore, Singapore; 24 Department of Urology, General Hospital of Heraklion, University of Crete, Heraklion, Greece; 25 Marmara University Pendik Training and Research Hospital, Istanbul, Turkey; 26 Department of Urology, IRCCS Fondazione Ca’ Granda Ospedale Maggiore Policlinico, Milan, Italy; 27 Department of Urology, Xinhua Hospital Affiliated to Shanghai Jiao Tong University School of Medicine, Shanghai, China; 28 Department of Urology, Aswan University, Aswan, Egypt; 29 Department of Urology, Guangdong Key Laboratory of Urological Diseases, The First Affiliated Hospital of Guangzhou Medical University, Guangzhou, China; 30 Department of Urology, Second Affiliated Hospital of Zhejiang University, Hangzhou, China; 31 Urology Unit, Department of Surgery, Hospital Sultanah Bahiyah, Kedah, Malaysia; 32 Department of Urology, Asklepios Hospital Barmbek, Hamburg, Germany; 33 Department of Urology, University Hospital Schleswig-Holstein, Campus Lübeck, Lübeck, Germany; 34 Institute of Kidney Diseases, Hayatabad Medical Complex Peshawar, Peshawar, Pakistan; 35 2nd Department of Urology, , National and Kapodistrian University of Athens, Athens, Greece; 36 Department of Urology, Hospital Clinic-University of Barcelona, Barcelona, Spain; 37 Hospital Nossa Senhora das Graças, Curitiba, Brazil; 38 Department of Medicine and Surgery, LUM University, Casamassima, Bari, Italy; 39 Urology Unit, Ecclesiastical Entity Regional General Hospital “F. Miulli”, Acquaviva delle Fonti, Bari, Italy

**Keywords:** Bladder stones, Cystolithotripsy, Endourology, Stone-free rate, Surgical outcomes

## Abstract

**Purpose:**

This registry aimed to establish a comprehensive global database to characterise contemporary real-world surgical approaches to bladder stones and to describe postoperative stone-free rate (SFR) and complications according to the surgical treatment route.

**Methods:**

The BLAST (Bladder Lithiasis Assessment of Surgical Treatment) registry prospectively enrolled 805 patients across 31 centres in 16 countries between May 2025 and February 2026. Patients underwent transurethral intervention (TI, *n* = 741), percutaneous suprapubic (PSI, *n* = 39), or open surgery (OI, *n* = 25). Postoperative SFR was graded using cross-sectional computer tomography, or ultrasonography, X-ray, or flexible cystoscopy, as Grade A (100% complete clearance), Grade B (single residual fragment), Grade C (multiple residual fragments). Perioperative complications were classified by the Clavien-Dindo criteria.

**Results:**

The cohort was predominantly male across all groups. TI patients were older (median 66 vs 60 and 59 years; *p* = 0.023) and presented with smaller stones (median 2.1 vs 3.0 cm in both other groups; *p* < 0.001). A history of urinary tract reconstruction was markedly more prevalent in the PSI cohort (23.1% vs 3.5% and 4.0%; *p* < 0.001). Median hospital stay was significantly shorter following TI (2 vs 3 days; *p* < 0.001), accompanied by a shorter duration of catheterisation (*p* < 0.001). Early complications within 48 h were most frequent after OI (48.0% vs 18.6% and 17.9%; *p* = 0.001), although major complications (Clavien-Dindo ≥ 3) remained rare and comparable across all modalities. No mortality was recorded. Grade A SFR was achieved in 95.5% of TI, 84.6% of PSI, and 100% of OI (*p* = 0.001). Reintervention for residual fragments was required significantly more often following PSI (10.3% vs 1.3% and 0%; *p* < 0.001).

**Conclusion:**

Transurethral cystolithotripsy was the predominant approach in this real-world international cohort and was associated with high stone clearance and shorter postoperative recovery. Percutaneous and open approaches were selectively used in patients with larger stone burdens, complex anatomy, prior urinary tract reconstruction, or specific urethral limitations. These findings describe contemporary treatment patterns and observed outcomes rather than comparative treatment effectiveness.

**Supplementary Information:**

The online version contains supplementary material available at https://doi.org/10.1007/s00345-026-06813-3.

## Introduction

Bladder stones account for approximately 5% of all urinary tract calculi; however, their clinical significance extends far beyond the stones themselves. They frequently serve as a hallmark of underlying lower urinary tract dysfunction and are associated with substantial, often underreported morbidity [1–4]. The incidence of vesical calculi is notably higher in developing nations, where the pathogenesis is frequently multifactorial. Common aetiological factors include bladder outlet obstruction (BOO), neurogenic bladder dysfunction, the presence of foreign bodies, prior bladder augmentation, recurrent urinary tract infections, upper tract stone migration, and underlying metabolic abnormalities [1–5].

According to the European Association of Urology (EAU) guidelines, bladder stones exhibit a strong male predominance, with a reported male-to-female ratio ranging from 4:1 to 10:1. The disease also demonstrates a bimodal age distribution, with a distinct peak in children from developing countries, largely driven by endemic dehydration, nutritional deficiencies, and dietary factors [1, 2].

Recent technological advancements in endourological equipment and laser lithotripsy platforms have substantially broadened the surgical armamentarium for both urolithiasis and benign prostatic obstruction (BPO). Consequently, vesical calculi of varying sizes, compositions, and quantities can now be managed highly effectively using minimally invasive approaches, either as isolated procedures or concurrently with the surgical treatment of the underlying pathology. Contemporary surgical management is highly individualised, tailored to specific stone characteristics, patient anatomy, and the available local surgical expertise [1–4, 6–10, 13].

Broadly, contemporary surgical interventions for bladder stones are classified into four main categories:

(A) transurethral intervention (TI);

(B) percutaneous suprapubic intervention (PSI);

(C) open intervention (OI); and.

(D) extracorporeal shock wave lithotripsy (ESWL).

Despite the widespread adoption of these approaches, large-scale, prospective, multicentre comparative data remain limited, and the optimal selection criteria for each modality in contemporary practice are not yet fully defined.

The primary objective of the BLAST (Bladder Lithiasis Assessment of Surgical Treatment) registry was to establish a comprehensive global database to characterise contemporary real-world surgical approaches to bladder stones and to describe postoperative stone-free rates according to the surgical treatment route. Secondary objectives were to document perioperative outcomes, complications, 30-day reintervention rates, and technical and technological variations in contemporary bladder stone surgery.

## Material and methods

### Patients and procedures

**Fig. 1:**
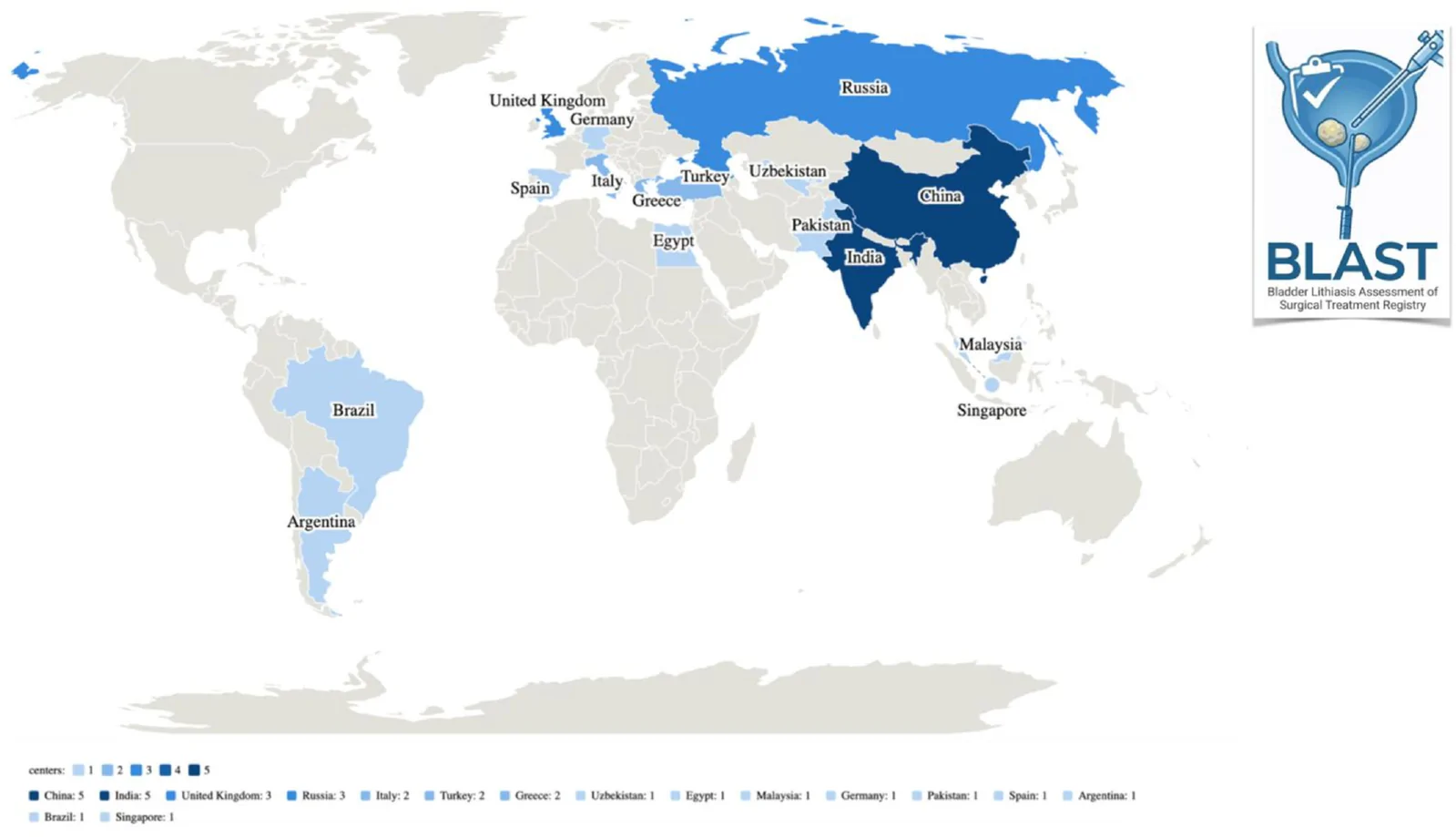
Heatmap of participating countries in BLAST registry

A prospective, multicentre, international registry was established to track the surgical management of bladder stones, incorporating data from 31 specialised centres across 16 countries (Fig. 1). Patients eligible for inclusion were adults who underwent an attempted surgical intervention for bladder stones between May 1, 2025, and February 15, 2026. The registry captured both patients undergoing isolated stone surgery and those requiring simultaneous concomitant urological procedures (e.g., prostate enucleation or resection, urethral dilation, or upper tract procedures). Exclusion criteria were strictly defined and included pregnant women, individuals with known active malignancies (both urological and non-urological), patients unable to complete a minimum of 6 weeks of structured postoperative follow-up, and cases with fundamentally incomplete medical records. All collected data were thoroughly anonymised and maintained within a secure, institutional review board-approved registry hosted at the Chinese University of Hong Kong (CRE-2021.684).

To accurately reflect real-world practice, there were no protocol-mandated limitations on the choice of surgical approach. Patients could be managed via endourological, percutaneous, or traditional open techniques. The surgical approach was decided case by case and there was no preference in a specific country. Operating surgeons autonomously selected their preferred access sheaths, scopes, and lithotripsy energy devices based on their individual expertise, clinical judgment, and institutional resources. Preoperatively, all patients were required to provide at least one urine culture; positive cultures were treated with targeted, sensitivity-guided antibiotic therapy in accordance with local antibiogram data, although a sterile preoperative culture was not strictly mandatory for inclusion.

Preoperative stone burden was quantified using available imaging modality (computed tomography [CT], ultrasonography, or plain X-ray radiography). The largest single stone diameter was recorded as the primary measure of stone burden. Where feasible, the total number of stones was also documented for patients presenting with multiple calculi.

### Follow-up and study outcomes

Baseline demographic parameters, comorbidities, and detailed operative characteristics were extracted from institutional electronic medical records. The primary outcome, postoperative stone-free rate (SFR), was determined using postoperative imaging (CT, ultrasonography, or plain X-ray) or direct visual inspection via flexible cystoscopy and performed 4–6 weeks after surgery. SFR was standardised according to the following grading criteria:

Grade A: 100% stone-free; zero residual fragments.

Grade B: single residual fragment of any size.

Grade C: multiple residual fragments of any size.

Defining Grade B as a single residual fragment of any size in bladder stone surgery ensures that even the smallest remnant is categorized as incomplete clearance, because the unique physiological environment of the urinary bladder guarantees that any retained fragment acts as a permanent nidus for stone regrowth if left untreated or not spontaneously expulsed.

Operative time was defined as the interval from the initial cystoscope insertion (or initial skin incision for open cases) to the final implementation of the chosen exit strategy (placement of a urethral catheter, suprapubic catheter, or decision for tubeless drainage). Secondary outcomes encompassed specific intraoperative findings, detailed perioperative complications classified by the Clavien-Dindo system [18, 19], and the incidence of postoperative sepsis, defined according to the Third International Consensus Definitions for Sepsis and Septic Shock (Sepsis-3) [16].

### Statistical analyses

Continuous variables were described using median and interquartile range, while categorical variables were described using absolute numbers and percentages. Variables were compared between endourological, percutaneous, and open interventions using the χ2 test or Fisher exact test for categorical parameters and the Kruskal–Wallis test for continuous variables. A subgroup analysis was performed for patients who had only bladder stone surgery.

All statistical analyses were performed using the R statistical language, version 4.4.2 (R Foundation for Statistical Computing, Vienna, Austria), with* p* < 0.05 indicating statistical significance.

## Results

### Overall population

A total of 805 patients were analysed, including 741 (92%) managed by transurethral endoscopic surgery (TI), 39 (4.8%) by percutaneous suprapubic surgery (PSI), and 25 (3.1%) by open surgery (OI). Supplementary Table 1 shows the number of patients enrolled by each country. The cohort was predominantly male, with no significant difference in sex distribution between groups. Patients undergoing transurethral treatment were older, whereas comorbidity profiles were otherwise broadly comparable. Stone burden varied significantly: the median largest stone diameter was 2.1 cm in the transurethral group versus 3.0 cm in both the percutaneous and open groups (*p* < 0.001). Prior bladder stone disease was more frequent in the percutaneous cohort, and previous urinary tract reconstruction was markedly overrepresented in this group (23.1% vs 3.5% and 4.0%; *p* < 0.001), indicating that percutaneous surgery was preferentially used in more complex anatomical settings (Table 1).

**Table 1:** Baseline characteristics

	Endoscopic transurethral intervention group (TI), Group 1 (*N* = 741)	Endoscopic Percutaneous suprapubic intervention (PSI), Group 2 (*N* = 39)	Open intervention (OI), Group 3 (*N* = 25)	*p*
Age	66 [55, 74]	60 [46, 70]	59 [52, 65]	**0.023**
Male sex	649 (90.0)	32 (82.1)	21 (84.0)	0.193
BMI, kg/m^2^	26.0 [23.1, 28.7]	26.0 [24.0, 28.4]	23.6 [22.0, 24.6]	**0.005**
Diabetes mellitus	174 (23.5)	9 (23.1)	3 (12.0)	0.408
Hypertension	311 (42.0)	14 (35.9)	5 (20.0)	0.072
Ischemic heart disease	105 (14.2)	8 (20.5)	4 (16.0)	0.537
Blood thinners (managed as per institutions protocol)	129 (17.4)	6 (15.4)	2 (8.0)	0.451
SAPT	69 (57.5)	5 (83.3)	2 (100)	
DAPT	5 (4.2)	0	0	
SAPT with NOAC	2 (1.7)	0	0	
NOAC	34 (28.3)	1 (16.7)	0	
VKA	7 (5.8)	0	0	
LMWH	3 (2.5)	0	0	
Largest stone diameter, cm	2.1 [1.5, 3.0]	3.0 [1.7, 3.8]	3.0 [2.2, 4.1]	**< 0.001**
Previous urolithiasis	202 (27.3)	14 (35.9)	6 (24.0)	0.461
Kidney	85 (11.5)	5 (12.8)	1 (4.0)	0.487
Ureter	52 (7.0)	2 (5.1)	3 (12.0)	0.563
Bladder	89 (12.0)	11 (28.2)	2 (8.0)	**0.01**
Previous surgery for urolithiasis	150 (20.2)	11 (28.2)	2 (8.0)	0.146
Previous urological surgery (non-urolithiasis)	126 (17.5)	10 (25.6)	6 (24.0)	0.326
History of urinary tract reconstruction	26 (3.5)	9 (23.1)	1 (4.0)	**< 0.001**
On IDC or CIC at time of surgery	208 (28.1)	13 (33.3)	9 (36.0)	0.549
Positive urine culture	181 (24.4)	14 (35.9)	10 (40.0)	0.066

*BMI* body mass index, *SAPT* single antiplatelet therapy, *DAPT* dual antiplatelet therapy, *NOAC* non-vitamin K anticoagulant oral anticoagulants, *VKA* vitamin K antagonists, *LMWH* low molecular weight heparin, *IDC* indwelling catheter, *CIC* clean intermittent catheterization

Bold values indicate results that reached statistical significance (p<0.05)

Operative characteristics and intraoperative strategies differed markedly by treatment route (Table 2). Rigid cystoscopy was the dominant access instrument (98.9%), while flexible endoscopic inspection at procedure completion was used more often in the percutaneous group (17.9% vs 2.8% and 4.0%; *p* < 0.001). Intraoperative complete clearance was achieved in 95.4% of transurethral cases, 82.1% of percutaneous cases, and 100% of open cases (*p* = 0.001). Fragmentation strategy varied substantially (p < 0.001): laser lithotripsy predominated in the transurethral group (57.5%), whereas mechanical lithotripsy was most common in the percutaneous group (80.0%). Suction-assisted devices were more frequently used in percutaneous procedures (30.8% vs 18.6% and 0%; *p* = 0.009). Median lithotripsy time was shortest for transurethral surgery (21 min, IQR 11–35) and significantly longer for both percutaneous and open approaches (34 and 35 min, respectively; *p* = 0.002). Despite these differences in fragmentation speed, the total operative time dedicated specifically to bladder stone treatment was statistically similar across all three groups (p = 0.104). Concomitant procedures were performed in approximately 40% of all cases, most frequently anatomical endoscopic enucleation of the prostate (AEEP; *n* = 127) or transurethral resection of the prostate (TURP; *n* = 86).

**Table 2:** Operative characteristics

	Endoscopic transurethral intervention group (TI), Group 1 (*N* = 741)	Endoscopic Percutaneous suprapubic intervention (PSI), Group 2 (*N* = 39)	Open intervention (OI), Group3 (*N* = 25)	*p*
Type of scope used				**< 0.001**
Rigid cystoscope	733 (98.9)	21 (87.5)	1 (100)	
Flexible cystoscope	3 (0.4)	0	0	
Rigid and flexible cystoscope	3 (0.4)	1 (4.2)	0	
24fr Nephroscope	0	2 (8.3)	0	
Semirigid ureteroscope	2 (0.3)	0	0	
General anaesthesia	383 (51.7)	12 (30.8)	11 (44.0)	**0.032**
Additional new stone seen on-table	56 (7.6)	6 (15.4)	2 (8.0)	0.212
Flexible scope used for inspection at end of operation	21 (2.8)	7 (17.9)	1 (4.0)	**< 0.001**
Intraoperative 100% SFR	707 (95.4)	32 (82.1)	25 (100)	**0.001**
Suction assisted device used (Shockpulse [Olympus, USA]; Trilogy [Electro Medical System, Switzerland], vacuum assisted aspiration)	138 (18.6)	12 (30.8)	0	**0.009**
Energy				**< 0.001**
Laser only	413 (57.5)	6 (17.1)	1 (50.0)	
Mechanical only	282 (39.3)	28 (80.0)	1 (50.0)	
Laser + mechanical	23 (3.2)	1 (2.9)	0	
Mechanical energy				**0.023**
Stone punch	9 (3.0)	0	0	
Forceps or Lithotrite	7 (2.3)	0	0	
Shockpulse (Olympus, USA)	30 (9.8)	8 (27.6)	1 (100)	
Trilogy (Electro Medical System, Switzerland)	13 (4.3)	0	0	
Lithoclast (Electro Medical System, Switzerland)	246 (80.7)	21 (72.4)	0	
Laser energy				**0.011**
Low power Holmium < 40 W	110 (25.2)	3 (42.9)	0	
High power Holmium > 100 W	150 (34.4)	0	1 (100)	
Holmium with MOSES (Lumenis, Israel)	51 (11.7)	0	0	
Thulium fiber laser (TFL) 40W and 60W	97 (22.2)	1 (14.3)	0	
Pulsed Thu:YAG	23 (5.3)	3 (42.9)	0	
Magneto (Quanta Spa system, Italy)	5 (1.1)	0	0	
Lithotripsy time, min	21 [11, 35]	34 [24, 45]	35 [35, 35]	**0.002**
Operation time for bladder stone, min	45 [25, 75]	47 [39, 73]	45 [40, 65]	0.104
Concomitant surgery	312 (42.3)	15 (39.5)	9 (37.5)	0.847
AEEP	126	0	1	
TURP	85	0	1	
URS and/or stent	28	1	1	
PCNL and/or PCN	3	10	0	
Urethral dilation	13	0	0	
Bladder neck incision	19	0	0	
Bladder biopsy (unconfirmed histology)	7	0	0	
Prostate biopsy	7	0	0	
Simple Prostatectomy	0	4	3	
Postoperative catheter	729 (98.4)	38 (97.4)	22 (88.0)	**0.001**
Urethral	705 (96.6)	10 (26.3)	16 (72.7)	
Suprapubic (new)	2 (0.3)	22 (57.9)	2 (9.1)	
Suprapubic (pre-existing)	8 (1.1)	3 (7.9)	1 (4.5)	
Urethral + suprapubic	15 (2.1)	3 (7.9)	3 (13.6)	
IDC removal on POD	1 [1, 3]	3 [1.5, 6.5]	21 [6, 21]	**< 0.001**

*AEEP* anatomical endoscopic enucleation of the prostate, *TURP* transurethral resectionof prostate, *URS* ureteroscopy, *PCNL* percutaneous nephrolithotomy, *PCN* percutaneous nephrostomy, *IDC* indwelling catheter, *POD* postoperative day

Bold values indicate results that reached statistical significance (p<0.05)

Postoperative recovery metrics strongly favoured the transurethral approach (Table 3). The median hospital length of stay was 2 days following transurethral treatment, compared to 3 days after both percutaneous and open surgery (*p* < 0.001). Similarly, the time to catheter removal was significantly shorter following transurethral surgery (median postoperative day 1 [IQR 1–3]) than after percutaneous (POD 3 [IQR 1.5–6.5]) or open procedures (POD 21 [IQR 6–21]; *p* < 0.001).

**Table 3:** Intraoperative and postoperative complications and stone-free rate

	Endoscopic transurethral intervention group (TI), Group 1 (*N* = 741)	Endoscopic Percutaneous suprapubic intervention (PSI), Group 2 (*N* = 39)	Open intervention (OI), Group 3 (*N* = 25)	*p*
Hospital stay, days	2 [1, 4]	3 [3, 5]	3 [2, 5]	**< 0.001**
On-table complications	12 (1.6)	1 (2.6)	2 (8.3)	0.054
Surgery abandoned	1 (0.1)	2 (5.3)	0	**< 0.001**
48-h complications, all	138 (18.6)	7 (17.9)	12 (48.0)	**0.001**
48-h complications, Clavien-Dindo ≥3	30 (4.0)	0	1 (4.0)	0.44
Sepsis within 48 h	5 (0.7)	0	0	0.805
6-week complications	15 (2.0)	2 (5.1)	2 (8.0)	0.078
Mortality	0	0	0	NA
Modality of stone-free rate evaluation, *n* (%)				**< 0.001**
CT scan	154 (20.8)	15 (38.5)	5 (20.0)	
Ultrasound	230 (31.0)	8 (20.5)	4 (16.0)	
X-rays and ultrasound	305 (41.2)	14 (35.9)	2 (8.0)	
Flexible cystoscopy	52 (7.0)	2 (5.1)	14 (56.0)	
Stone-free rate grading				**0.001**
A	708 (95.5)	33 (84.6)	25 (100)	
B	22 (3.0)	2 (5.1)	0	
C	11 (1.5)	4 (10.3)	0	
Reintervention for residual fragment	10 (1.3)	4 (10.3)	0	**< 0.001**

*TI* transurethral intervention (1), *PSI* percutaneous suprapubic intervention (2), *OI* open intervention (3), *EI* ESWL intervention (4)

Bold values indicate results that reached statistical significance (p<0.05)

From a safety perspective, intraoperative complications were uncommon across the entire cohort, although they were numerically more frequent in the open surgery group (8.3% vs 1.6% and 2.6%; *p* = 0.054). Early postoperative complications (within 48 h) were significantly more frequent following open surgery (48.0% vs 18.6% for TI and 17.9% for PSI; *p* = 0.001). However, severe major complications (Clavien-Dindo grade ≥ 3) were exceptionally rare and statistically comparable between all groups (4.0% for TI, 0% for PSI, 4.0% for OI; *p* = 0.440). Postoperative sepsis was exceedingly rare (0.7% in the TI group), and no perioperative mortality was observed in the entire cohort. Modified sheaths or technical modifications were noted in patients from nine centers across four countries: India, Russia, China, and Turkey*.*

At final follow-up, imaging-confirmed complete stone-free status (Grade A) was achieved in 95.5% of transurethral cases, 84.6% of percutaneous cases, and 100% of open cases (*p* = 0.001). However, the groups showed a statistically significant different distribution patterns across the four diagnostic methods for SFR evaluation (*p* < 0.001): CT scan (20.8% vs 38.5% vs. 20.0%), Ultrasound (31.0% vs 20.5% vs. 16.0%), X-rays plus ultrasound (41.2% vs 35.9% vs. 8.0%), and Flexible cystoscopy (7.0% vs 5.1% vs. 56.0%), respectively. The need for subsequent reintervention to manage residual stone fragments was significantly more frequent following percutaneous surgery compared to transurethral or open treatment (10.3% vs 1.3% and 0%, respectively; *p* < 0.001).

### Subgroup analysis of patients having only bladder stone surgery

Among 463 patients treated without a concomitant procedure, baseline characteristics were similar for median age, gender distribution, and comorbidity but differed significantly for stone characteristics and previous surgery (Supplementary Table 2). Patients undergoing OI presented with larger stones (median 3.0 cm vs. 2.0–2.5 cm, *p* = 0.029) and had lower rates of previous urolithiasis, prior urological surgery, and prior urinary tract reconstruction compared with the PSI group, which had markedly higher rates of prior surgical history (most strikingly a history of urinary tract reconstruction: 30.4% in PSI vs. 3.8% in TI and 6.7% in OI, p < 0.001). Operatively, procedure time was shortest with TI (median 30 min) and longest with PSI (50 min) and OI (40 min) (*p* < 0.001) (Supplementary Table 3). Intraoperative stone-free rate was highest for OI (100%) and lowest for PSI (87.0%) versus TI (97.6%, *p* = 0.008). Suprapubic catheter placement and end-of-procedure flexible-scope inspection were both far more common after PSI. Time to catheter removal was substantially longer after OI (median 21 days) than TI or PSI (1–2 days, *p* < 0.001). Postoperatively (Supplementary Table 4), hospital stay was longest after PSI (median 3 days) versus TI and OI (2 days each, *p* = 0.001). Notably, 48-h complication rates were highest after OI (60.0%), intermediate after TI (22.4%), and lowest after PSI (4.3%, *p* < 0.001), while on-table complications and abandoned surgeries were more frequent in the PSI group. Stone-free status at follow-up (grade A) was achieved in 100% of OI patients versus 95.5% of TI and 78.3% of PSI patients (*p* < 0.001), and reintervention for residual fragments was most common after PSI (17.4% vs. ≤ 0.9% in the other groups, *p* < 0.001).

## Discussion

This large, prospective, multicentre international registry provides contemporary real-world data on the surgical management of bladder stones and demonstrates a predominant use of the transurethral approach across participating centres. Transurethral treatment was associated with high stone clearance and shorter postoperative recovery in our cohort. However, these findings should be interpreted primarily as descriptive, as treatment allocation was non-randomised and the three treatment groups differed substantially in patient characteristics, stone burden, and anatomical complexity. In particular, the preferential use of percutaneous and open approaches in patients with larger stones and complex anatomy introduces substantial confounding by indication and precludes attributing differences in outcomes directly to the surgical access route.

Current European Association of Urology guidelines recommend transurethral cystolithotripsy whenever anatomically feasible, recognising that while bladder stones account for only a small minority of all urinary tract calculi, they frequently arise in the complex clinical context of bladder outlet obstruction, neurogenic bladder dysfunction, chronic bacteriuria, long-term indwelling catheters, altered reconstructive anatomy, or subsequent to migration from the upper urinary tract [1, 2]. The demographic and clinical profile of the present BLAST cohort closely mirrors this established epidemiological pattern, being predominantly male and older, with a substantial burden of associated urological pathology and a high frequency of concomitant surgical procedures. The true clinical value of the transurethral approach lies in its unique ability to permit the definitive, simultaneous treatment of both the stone burden and the underlying contributing outlet pathology during a single hospital admission, and frequently within the same operative session. This integrated, single-stage strategy is particularly relevant and beneficial for men with benign prostatic obstruction (BPO), for whom recurrent urinary stasis, chronic infection, and incomplete bladder emptying act as the central physiological drivers of de novo bladder stone formation [1, 2, 6].

However, the direct comparison between access routes must be interpreted carefully through the critical lens of case selection. Patients managed percutaneously or via open surgery in this registry presented with significantly larger stone burdens, and the percutaneous cohort exhibited a markedly higher prevalence of previous complex urinary tract reconstruction, indicating strong confounding by indication. In this context, the lower complete stone-free rate (84.6%) and the higher subsequent reintervention rate (10.3%) observed following percutaneous surgery should not be simplistically interpreted as evidence of procedural inferiority. Instead, these outcomes directly reflect the deliberate selection of PSI for a highly complex cohort in which standard transurethral instrumentation may have been technically impossible, highly inefficient, or potentially hazardous [1, 2]. Contemporary comparative studies similarly suggest that both percutaneous and transurethral techniques are capable of achieving excellent clearance rates when appropriately matched to the patient’s specific stone size and anatomy [7–10].

Open surgery, while increasingly rare in modern practice, remains the most direct and reliable route to guarantee complete stone removal and may still be entirely justified for exceptionally large stones or when simultaneous open pelvic reconstruction is mandatory. Nevertheless, its inherently greater surgical invasiveness continues to impose a significant penalty in terms of postoperative recovery time and early complication rates [9, 11]. The technical data captured in the present study reinforce a broader, fundamental principle: success in bladder stone surgery depends far less on the pursuit of a single “superior” energy technology than on correct, individualised route selection and the meticulous, complete evacuation of all generated fragments. Laser lithotripsy predominated in the transurethral group, whereas mechanical fragmentation and suction-assisted evacuation devices were far more frequent in the percutaneous cohort, reflecting a practical adaptation to working channel size, total stone burden, and the critical need for rapid fragment extraction [2, 10–12]. Although enthusiasm for newer platforms such as the thulium fiber laser (TFL) is rapidly increasing globally, the strongest evidence to date suggests that laser choice primarily influences intraoperative fragmentation efficiency rather than the final absolute stone clearance rate [11, 12].

An equally important long-term implication of this registry concerns recurrence prevention. A successful cystolithotripsy does not, by itself, correct the underlying biological and functional environment that permitted the stone to form. This is especially relevant for men with BPO and for patients managing neurogenic bladders or relying on catheter-dependent drainage, all of whom carry a substantial long-term risk of stone recurrence if the fundamental mechanism of urinary stasis or chronic infection remains unaddressed [1, 5, 6, 13]. Emerging literature increasingly supports a selective approach to simultaneous bladder outlet surgery: combined treatment appears to significantly reduce future stone recurrence and reintervention in many men, yet not all patients require immediate concurrent prostate surgery. Critical factors—such as post-void residual volume, intravesical prostatic protrusion, symptom burden, and the expected degree of de-obstruction—must carefully inform the shared decision-making process [6, 14, 15, 17]. While the present registry cannot definitively resolve the debate surrounding simultaneous surgery, the exceptionally high frequency of concomitant procedures (> 40%) strongly suggests that contemporary surgeons already recognise bladder stones as a marker of broader lower urinary tract pathology rather than an isolated, episodic event.

From a safety perspective, the results of this global study are highly reassuring. Although early minor postoperative complications were significantly more frequent following open surgery, severe Clavien-Dindo grade ≥3 events were remarkably uncommon across all three groups, postoperative sepsis was exceedingly rare, and no perioperative mortality occurred in this large cohort. These data strongly support the view that all three surgical approaches can be delivered safely in experienced urological centres when carefully matched to the right patient [16, 18, 19].

That said, the subgroup analysis restricted to patients undergoing bladder stone surgery alone offers an important lens on the overall cohort findings, revealing that several differences observed in the full registry were substantially attenuated, or even reversed, once concomitant procedures were excluded. Most notably, operative time dedicated specifically to bladder stone treatment appeared statistically similar across the three surgical routes in the overall cohort (45, 47, and 45 min for TI, PSI, and OI), yet became markedly and significantly divergent once concomitant-procedure patients were removed (30, 50, and 40 min). This discrepancy is best explained by the high frequency of combined bladder outlet surgery performed alongside transurethral cystolithotripsy, which substantially inflated total procedure time in a large proportion of TI cases and masked the comparatively rapid isolated stone clearance achievable by this route. A similar confounding effect likely underlies the age difference seen in the overall cohort (TI patients were significantly older), which disappeared entirely in the bladder-stone-only subgroup, consistent with older men with symptomatic benign prostatic enlargement preferentially undergoing combined TI-AEEP/TURP procedures. Perhaps most strikingly, the pattern of early postoperative complications diverged between analyses: in the overall cohort, complication rates were comparable between TI and PSI (18.6% vs 17.9%), with open surgery clearly highest (48.0%), whereas in the bladder-stone-only subgroup, PSI complication rates fell to the lowest of the three groups (4.3%), TI rose to 22.4%, and OI increased further still (60.0%). Taken together, these observations suggest that concomitant bladder outlet surgery acts as an important effect modifier that can obscure, and in some cases invert, apparent differences in early morbidity between bladder stone treatment routes, underscoring the need for cautious, stratified interpretation whenever registries combine isolated and combined surgical cohorts. Given the small numbers of patients undergoing percutaneous (n = 23) and open (n = 15) bladder-stone-only surgery, however, these subgroup findings, particularly the complication-rate reversal, should be regarded as hypothesis-generating rather than definitive, and warrant confirmation in larger, dedicated cohorts.

This study has several limitations that should be acknowledged. First, the pragmatic, observational design introduces inherent selection bias, with marked numerical imbalance between treatment groups, non-randomised treatment allocation, centre-level heterogeneity in instrumentation and perioperative protocols, and non-uniform modalities used for postoperative stone-free status assessment. Second, the registry did not capture granular technical details regarding concomitant procedures, including the approach, sequence, and perioperative management of combined surgeries. These factors may influence outcomes and their attribution to the stone treatment route, and we were unable to perform a patient-level or subgroup analysis of these uncommon combinations**.** Third, the observation of technical modifications across nine centers in four countries highlights global adaptability and resource-driven innovation, though these were not systematically captured. Consequently, these findings must be interpreted within the context of these methodological constraints. Nevertheless, these same features—the real-world, multicentre, prospective design—significantly enhance the external validity of the study, accurately capturing how bladder stone surgery is performed across diverse global practice settings. Future risk-adjusted analyses should aim to evaluate not only immediate stone clearance rates but also long-term recurrence, optimal strategies for outlet management, and patient-specific predictors of clinical benefit from each surgical route. Finally, residual fragments assessment was performed using different modalities across the groups and this could have influenced the final SFR.

## Conclusion

Transurethral cystolithotripsy was the predominant surgical approach in this prospective international real-world cohort and was associated with high stone clearance, shorter catheterisation, and shorter hospital stay. Percutaneous and open approaches were selectively employed in patients with larger stone burdens, prior urinary tract reconstruction, and other complex anatomical or clinical features. Given the substantial differences in patient selection and the non-randomised nature of treatment allocation, observed differences in outcomes between approaches should not be interpreted as evidence of comparative treatment effectiveness. Overall, these findings provide contemporary real-world insight into the selection and outcomes of different surgical approaches to bladder stones and support an individualised treatment strategy based on stone burden, patient anatomy, associated lower urinary tract pathology, and local surgical expertise.

## Supplementary Information

Below is the link to the electronic supplementary material.

Supplementary Material 1

## Data Availability

No datasets were generated or analysed during the current study.
