# Supplementary material for: BLAST—bladder lithiasis assessment of surgical treatment in real-world practice. A global prospective multicentre registry by EAU Endourology
Source: World J Urol. 2026 Oct 13;44(1):745. doi: 10.1007/s00345-026-06813-3 (PMC13661746; doi:10.1007/s00345-026-06813-3)
Supplement: Supplementary file 1 [file 345_2026_6813_MOESM1_ESM.docx]

**Supplementary Table 1.** Number of patients enrolled by each country (number of countries= 16).

| **Country** | **Number of patients** |
| --- | --- |
| Spain | 20 |
| Italy | 53 |
| Germany | 20 |
| India | 170 |
| Russia Federation | 85 |
| Argentina | 20 |
| China | 148 |
| United Kingdom | 67 |
| Egypt | 21 |
| Greece | 44 |
| Malaysia | 20 |
| Pakistan | 20 |
| Singapore | 23 |
| Türkiye | 50 |
| Uzbekistan | 24 |
| Brazil | 20 |
| **Total** | 805 |

# **Supplementary Table 2.** Baseline characteristics of patients having only bladder stone surgery.

|  | Endoscopic transurethral intervention group (TI)  Group1 (N=425) | Endoscopic Percutaneous suprapubic intervention (PSI)  Group2 (N=23) | Open intervention (OI)  Group3 (N=15) | p |
| --- | --- | --- | --- | --- |
| Age | 63 [52, 72] | 58 [43, 65] | 60 [44, 67] | 0.15 |
| Male sex | 364 (86.1) | 16 (69.6) | 12 (80.0) | 0.083 |
| BMI, kg/m^2^ | 25.0 [22.9, 28.0] | 26.0 [23.9, 28.0] | 23.5 [20.6, 24.0] | **0.008** |
| Diabetes mellitus | 98 (23.1) | 6 (26.1) | 2 (13.3) | 0.633 |
| Hypertension | 155 (36.5) | 8 (34.8) | 3 (20.0) | 0.423 |
| Ischemic heart disease | 43 (10.1) | 4 (17.4) | 2 (13.3) | 0.511 |
| Blood thinners  (managed as per institutions protocol) | 48 (11.3) | 1 (4.3) | 1 (6.7) | 0.505 |
| SAPT | 29 (63.0) | 1 (100) | 1 (100) |  |
| DAPT | 4 (8.7) | 0 | 0 |  |
| NOAC | 11 (23.9) | 0 | 0 |  |
| VKA | 2 (4.3) | 0 | 0 |  |
| Largest stone diameter, cm | 2.0 [1.5, 2.9] | 2.5 [1.5, 3.1] | 3.0 [2.1, 4.0] | **0.029** |
| Previous urolithiasis | 107 (25.2) | 11 (47.8) | 2 (13.3) | **0.029** |
| Kidney | 43 (10.1) | 5 (21.7) | 0 | 0.084 |
| Ureter | 25 (5.9) | 1 (4.3) | 1 (6.7) | 0.945 |
| Bladder | 53 (12.5) | 9 (39.1) | 1 (6.7) | 0.001 |
| Previous surgery for urolithiasis | 85 (20.0) | 9 (39.1) | 1 (6.7) | **0.035** |
| Previous urological surgery (non-urolithiasis) | 65 (15.4) | 9 (39.1) | 3 (20.0) | **0.011** |
| History of urinary tract reconstruction | 16 (3.8) | 7 (30.4) | 1 (6.7) | **<0.001** |
| On IDC or CIC at time of surgery | 119 (28.0) | 9 (39.1) | 5 (33.3) | 0.477 |
| Positive urine culture | 108 (25.4) | 7 (30.4) | 7 (46.7) | 0.167 |

BMI – body mass index; SAPT - single antiplatelet therapy; DAPT – dual antiplatelet therapy; NOAC – non-vitamin K anticoagulant oral anticoagulants; VKA – vitamin K antagonists; LMWH – low molecular weight heparin; IDC – indwelling catheter; CIC – clean intermittent catheterization

# **Supplementary Table 3**. Operative characteristics of patients having only bladder stone surgery.

|  | Endoscopic transurethral intervention group (TI)  Group1 (N=425) | Endoscopic Percutaneous suprapubic intervention (PSI)  Group2 (N=23) | Open intervention (OI)  Group3 (N=15) | p |
| --- | --- | --- | --- | --- |
| Type of scope used | 422 (99.3) | 19 (86.4) | 0 |  |
| Rigid cystoscope | 1 (0.2) | 0 | 0 |  |
| Flexible cystoscope | 0 | 1 (4.5) | 0 |  |
| Rigid and flexible cystoscope | 0 | 2 (9.1) | 0 |  |
| 24fr Nephroscope | 2 (0.5) | 0 | 0 |  |
| Semirigid ureteroscope | 422 (99.3) | 19 (86.4) | 0 |  |
| General anaesthesia | 195 (45.9) | 7 (30.4) | 3 (20.0) | 0.055 |
| Additional new stone seen on-table | 29 (6.8) | 3 (13.0) | 1 (6.7) | 0.527 |
| Flexible scope used for inspection at end of operation | 4 (0.9) | 6 (26.1) | 0 | **<0.001** |
| Intraoperative 100% SFR | 415 (97.6) | 20 (87.0) | 15 (100) | **0.008** |
| Suction assisted device used (Shockpulse [Olympus, USA]; Trilogy [Electro Medical System, Switzerland], vacuum assisted aspiration) | 94 (22.1) | 7 (30.4) | 0 | 0.074 |
| Energy |  |  |  | NA |
| Laser only | 176 (42.0) | 5 (21.7) | 0 |  |
| Mechanical only | 225 (53.7) | 17 (73.9) | 0 |  |
| Laser + mechanical | 18 (4.3) | 1 (4.3) | 0 |  |
| Mechanical energy |  |  |  | NA |
| Stone punch | 4 (1.6) | 0 | 0 |  |
| Forceps or Lithotrite | 5 (2.1) | 0 | 0 |  |
| Shockpulse (Olympus, USA) | 21 (8.6) | 7 (38.9) | 0 |  |
| Trilogy (Electro Medical System, Switzerland) | 10 (4.1) | 0 | 0 |  |
| Lithoclast (Electro Medical System, Switzerland) | 203 (83.5) | 11 (61.1) | 0 |  |
| Laser energy |  |  |  | NA |
| Low power Holmium <40W | 79 (40.7) | 2 (33.3) | 0 |  |
| High power Holmium >100W | 48 (24.7) | 0 | 0 |  |
| Holmium with MOSES (Lumenis, Israel) | 35 (18.0) | 0 | 0 |  |
| Thulium fiber laser (TFL) 40W and 60W | 26 (13.4) | 1 (16.7) | 0 |  |
| Pulsed Thu:YAG | 5 (2.6) | 3 (50.0) | 0 |  |
| Magneto (Quanta Spa system, Italy) | 1 (0.5) | 0 | 0 |  |
| Lithotripsy time, min | 18 [10, 32] | 30 [24, 40] | NA | NA |
| Operation time for bladder stone, min | 30 [20, 52] | 50 [40, 75] | 40 [38, 56] | **<0.001** |
| Postoperative catheter | 416 (97.9) | 23 (100) | 13 (86.7) | **0.015** |
| Urethral | 410 (98.3) | 6 (26.1) | 10 (76.9) |  |
| Suprapubic (new) | 1 (0.2) | 12 (52.2) | 2 (15.4) |  |
| Suprapubic (pre-existing) | 4 (1.0) | 2 (8.7) | 1 (7.7) |  |
| Urethral + suprapubic | 2 (0.5) | 3 (13.0) | 0 |  |
| IDC removal on POD | 1.0 [1.0, 2.0] | 2.0 [0.2, 5.0] | 21 [21, 21] | **<0.001** |

IDC – indwelling catheter; POD – postoperative day

# **Supplementary Table 4.** Intraoperative and postoperative complications and stone-free rate of patients having only bladder stone surgery.

|  | Endoscopic transurethral intervention group (TI)  Group1 (N=425) | Endoscopic Percutaneous suprapubic intervention (PSI)  Group2 (N=23) | Open intervention (OI)  Group3 (N=15) | p |
| --- | --- | --- | --- | --- |
| Hospital stay, days | 2 [1, 3] | 3 [2, 3.5] | 2 [2, 5] | **0.001** |
| On-table complications | 3 (0.7) | 1 (4.3) | 1 (6.7) | **0.027** |
| Surgery abandoned | 1 (0.2) | 2 (8.7) | 0 | **<0.001** |
| 48-hour complications, all | 95 (22.4) | 1 (4.3) | 9 (60.0) | **<0.001** |
| 48-hour complications, Clavien-Dindo ≥3 | 13 (3.1) | 0 | 0 | 0.55 |
| Sepsis within 48 hours | 0 | 0 | 0 | NA |
| 6-week complications | 5 (1.2) | 2 (8.7) | 0 | **0.014** |
| Mortality | 0 | 0 | 0 | NA |
| Stone-free rate grading |  |  |  | **<0.001** |
| A | 406 (95.5) | 18 (78.3) | 15 (100) |  |
| B | 12 (2.8) | 1 (4.3) | 0 |  |
| C | 7 (1.6) | 4 (17.4) | 0 |  |
| Reintervention for residual fragment | 4 (0.9) | 4 (17.4) | 0 | **<0.001** |
